# Petroleum in Pesticides: A Need to Change Regulatory Toxicology

**DOI:** 10.3390/toxics10110670

**Published:** 2022-11-06

**Authors:** Gérald Jungers, Florence Portet-Koltalo, Julie Cosme, Gilles-Eric Seralini

**Affiliations:** 1 Network on Risks, Quality and Sustainable Environment and Faculty of Sciences, University of Caen Normandy, 14032 Caen, France; 2 Laboratory UMR CNRS 6014 COBRA, University of Rouen Normandy, 27930 Evreux, France

**Keywords:** polycyclic aromatic hydrocarbons, acceptable daily intake, pesticides, petroleum residues, formulants

## Abstract

Toxicological investigations of pesticides largely focus on the declared active ingredient, which constitutes only between a few percent to around 50% of the total formulation. The complete formulations are unknown. For each declared active ingredient, there are dozens or hundreds of formulations. We demonstrate that petroleum has always been and is still always in pesticides. Gas chromatography and mass spectrometry (GC-MS) were applied for 24 pesticides. The measured compounds were the 16-priority polycyclic aromatic hydrocarbons (PAHs). The ratio of the PAHs to the threshold of toxicity was from 2.16 to 8288 times. The levels and distribution of PAHs per pesticide were different. Petroleum residues appear to be a waste product. The declared active component is taken alone for toxicity calculations, such as the acceptable daily intake (ADI). The PAHs with 2–3 cycles are more represented in pesticides than those with 4–6 cycles, which underlines that the petroleum residues appear to come mainly from crude unburned material. The ADI should be divided by 1000 if it is considered that petroleum residues amplify the toxicity by 1000. The admixture of PAHs in pesticides can be highly carcinogenic or toxic in the long term, even more than the declared active ingredient itself.

## 1. Introduction

Currently, a pesticide active ingredient is authorised for marketing essentially based on tests for impacts on health and the environment commissioned by the industry applicant, which provides the data to the regulatory agencies. The main object of toxicity for a pesticide is the declared active ingredient, which, however, constitutes the minority of the ingredients that make up pesticide formulations. The complete formulation is unknown to the scientific community; it is kept hidden as an industrial secret. For each pesticide active ingredient, there are dozens or hundreds of formulations. Long-term tests on formulations for mammalian toxicology are not performed, or, if they are, they are not published.

The use of pesticides has been amplified in the world, especially since the second world war [1]. They were industrially developed before or during the war, often as toxic gases or explosives [2]. Nitrates and phosphates used today as fertilizers were developed from fossil fuels and were originally used as explosives. In general, pesticides are synthesized from petroleum [1,2,3] and their manufacturing has been linked to carbochemistry or petrochemistry [4].

Since then, petroleum and petroleum by-products have been used to make pesticides, as well as the detergents and formulants contained in pesticide formulations [1,5,6]. In fact, this is why the concept of active ingredient is only decided by the self-declaration of the manufacturer. The declared active ingredient constitutes only between a few percent and around 50% of the commercialized pesticide. The remaining formulants include a large list of products, which are described by various names of compounds that are not always clear scientifically and overlap one another. Again, they are named by the manufacturer. These compounds include additives, adjuvants, co-formulants, diluents, dispersants, emolients, emulsifiers, humectants, wetting agents, inerts, surfactant, and tensioactive agents. Since most of these products may not be declared, there is always scientific confusion about their real nature [3]. Historically, these products may also have been synthesized from petroleum; in addition, extracts from petroleum have also been used as pesticides (Table 1).

Petroleum has been used as a pesticide since 1787 [7]. Since 1954, for instance, the company Esso and others, originally known as the Seven Sisters oil companies, promoted the use of petroleum residues in pesticides [1], as demonstrated in Table 1.

Unrefined petroleum is usually called crude oil and is a complex mixture of saturated (paraffinic) and aromatic hydrocarbons (composed of C and H atoms); it also contains a low percentage of sulphur, traces of metals (for instance, arsenic), and nitrogen and oxygen compounds in resin or asphaltene fractions [25,26,27]. Treatment mainly consists of cracking and/or fractional distillation of crude oil. High temperatures cracking break long chain hydrocarbons (HC) into shorter ones and generate mainly unsaturated olefins. Distillation does not modify HC structures and consists of separating them into different fractions as a function of their boiling points. In a range of low temperatures, a light oil cut (giving gasoline) is obtained; middle distillate gives diesel or kerosene; wide-cut oil gives waxes or lubricating oils; and finally, at higher temperature ranges, residual oil produces asphalts. So refined petroleum products are derived from crude oils through processes such as cracking and distillation [25,26,27,28,29].

Polycyclic aromatic hydrocarbons (PAHs) are those HCs that have at least 2 fused aromatic rings. Among hundreds of possible structures, US-EPA distinguished 16 priority PAHs which are dominant in environment and studied here. It is now well documented that aromatic HCs are more toxic for living organisms than paraffinic ones, and consequently a majority of studies on the toxicological impact of HCs on the environment or organisms focuses on mono- or poly-aromatic HCs. PAHs are more or less present in crude oil or refined fractions. PAHs can be divided into low molecular weight (LMW) PAHs, having 2 or 3 condensed aromatics rings, whereas higher molecular weight (HMW) PAHs present 4 to 6 fused rings. The higher mass PAHs are generally minor contributors to crude oil or refined petroleum products. Lighter cuts mainly contain single ring aromatic HCs (benzene) and 2-ring naphthalene (and their methylated derivatives), and the content of PAHs increases in heavier crudes. HMW PAHs are present in significant amount only in higher fractions such as asphalts, whereas non-aromatic HCs are naturally degraded by high temperatures [25]. Numerous studies have demonstrated that the composition of PAHs in the environment can illustrate their petrogenic or pyrogenic sources. Petrogenic PAHs derive from petroleum inputs, whereas pyrogenic PAHs derive from incomplete combustion processes. The presence of high proportions of HMW PAHs generally indicates petroleum combustion–that is, transformation of carbonaceous compounds at high temperatures [26]. With the exception of asphalts, a majority of LMW PAHs indicate crude or refined petroleum sources without combustion or high temperature thermal processes [29].

In order to be more specific in the prediction of PAHs’ origins, several diagnostic ratios between PAH are given to identify pyrolytic and petrogenic sources in the environment. They are mainly applied to atmospheric deposits in soil or sediment samples [25,26,27,28,29]. For example, when the 16-priority PAHs only are considered, the calculation of the sum of LMW PAHs (2–3 aromatic rings) to the sum of HMW PAHs (4–6 aromatic rings) reveals a pyrogenic source if the ratio is < 1 or a petrogenic source if > 1 [29]. Considering isomeric ratios, PAHs of molecular mass 178 [Phenanthrene (PHEN), Anthracene (ANT), 202 (Fluoranthene (FLT), and Pyrene (PYR)] are commonly used to distinguish between unburned petroleum or combustion sources [27]. These ratios have never been used to characterize the possible source of PAHs present in pesticide formulations.

The purpose of this work is to demonstrate that petroleum has always been in the composition of pesticides (Table 1), even in recent formulations, and to investigate their possible sources. It will be shown that this knowledge has declined to the point where it is no longer known, or only poorly known, to the scientific world. In addition, this study shows that the nature and levels of PAHs in pesticides are extremely variable, from one trademark to another, and from one batch or lot to another, which is abnormal. Hence, it might be necessary in the future to take into account the presence of petroleum products in pesticides for long-term toxicity assessments and also to lower the toxicity thresholds.

## 2. Materials and Methods

### 2.1. Materials and Chemicals

We investigated herbicides that have been available on the French, German and Polish markets for gardeners or farmers between the end of the 20th century to 2019. They were selected from our pesticide university library or were gifts from users. Each was carefully numbered A-W and their respective contents, in terms of declared active principles and specifications, were listed (Table 2). To ensure the accuracy and reproducibility of the data, in particular for adequate replications, all quantifications measurements and their coefficients of variations were performed in laboratories accredited by COFRAC, the French accreditation body, or by the University of Rouen-Normandy (France).

Toluene and cyclohexane (HPLC grade, purity > 99.9%), technical acetone (purity > 95%) were provided by Fisher Scientific (Illkirch, France). Magnesium sulfate (purity > 98%) was furnished by Sigma Aldrich-Merck France (St Quentin Fallavier, France). Perdeuterated PHEN and PER (internal standards, IS for GC-MS), perdeuterated FLT and B[*a*]PYR (surrogate standards, SS) were provided by Sigma-Aldrich. Solutions containing the 16-priority PAHs, defined by US-EPA (2000 mg/L) were also furnished by Sigma-Aldrich.

Teflon PTFE filters were furnished by Phenomenex, (le Pecq, France) and solvents were evaporated with a MiVac duo concentrator (Genevac, Ipswich, United Kingdom). Pure deionized water was produced with a Smart2PureSamples device from Thermo Scientific (Montigny le Bretonneux, France). All the herbicides and chemical standards were stored in a freezer (4 °C).

### 2.2. Analyses of PAHs

Two different methods were applied to analyze the 16 priority PAHs in 24 liquid pesticide formulations. First, the internationally normalized method DIN 38407-39 was applied (method A), using gas chromatography coupled to mass spectrometry (GC-MS) for PAH measures. PAHs in water were extracted with cyclohexane. The extract was concentrated by evaporation. The PAHs were then separated by gas chromatography (GC) on capillary columns with suitable stationary separation phases and identified and quantified by MS. In detail, after adding a mixture of isotope-labelled standards to the samples, the extracts obtained by liquid/liquid extraction were dried with anhydrous sodium sulphate and concentrated prior to analysis by gas chromatography with mass spectrometer (GC/MS). Chromatographic separation was performed on a Varian VF-Xms column. Quantification was done by isotope dilution.

The measured compounds were the 16 priority PAHs (Table 3). 

Second (method B), some pesticide formulations were sonicated to homogenize them before analysis. They were then diluted by a factor of 100 or 1000 to avoid foaming during the liquid-liquid extraction (LLE). Indeed, herbicide formulations are in fact aqueous emulsions that tend to form more or less foam, depending on additives in their compositional. LLE consisted first of adding 25 mL cyclohexane into the diluted aqueous pesticide formulation. The two SS were added (10 µL of 100 mg/L solutions) to measure possible PAHs loss during the extraction process. Then the mixture was shaken for 20 min and decanted for 10 to 30 min, until the foam layer decreased. The organic phase was recovered, and the aqueous phase was put in contact for 20 min again, by stirring with 20 mL cyclohexane. After decantation, the two organic phases were combined, 4 to 5 g magnesium sulfate was added and the mixture was filtered through a 0.2 µm PTFE filter After addition of 60 µL octanol (solvent keeper), the organic phase was evaporated 2h 15 min at 45 °C under 30 mbar with a MiVac duo concentrator. Samples were reconstituted in 1 mL toluene, with an addition of 10 µL of the two IS (100 mg/L) for better quantification reliability. The mean recoveries of SS after LLE were in the range 77.1–106.4% (mean 90.4%) for perdeuterated FLT, 74.4–109.5% (mean 95.6%) for perdeuterated B[a]PYR. That shows that PAHs were not lost during the extraction process. Thereafter, 1 µL of extract was injected (split less mode, 285 °C) into a gas chromatograph (6850 series, Agilent), coupled with a mass spectrometer (5975C series). The separation was performed using a 60 m × 0.25 mm i.d. DB-5MS capillary column (0.25 µm film thickness; J&W Scientific, Agilent, France), with helium as the carrier gas (1.4 mL/min). The oven temperature was programmed: 60 °C (1.2 min), increased to 190 °C (40 °C/min), then to 240 °C (4 °C/min) and to 305 °C (6 °C/min), for 12 min. The transfer line temperature was 300 °C and the MS detector operated at 70 eV. Quantification was based on selected ion monitoring to improve sensitivity. Calibration curves were established using 6 levels of concentrations, from 0.1 to 3 mg/L, using the internal calibration methodology (in relation to the two IS), and all the determination coefficients were >0.990. All the pesticide formulations were analysed in duplicate. The GC-MS instrumental limits of detection (LODs) and quantification (LOQs) were estimated based on signal-to-noise ratios of 3:1 and 10:1, respectively. They were in the range of 0.44–36 µg/L and 1.47–120 µg/L for LODs and LOQs, respectively. In general, procedural LOQs were <2 µg/L but were higher when dilution factors were applied. Procedural blank samples (n = 4) made from pure water and following the entire analytical procedure, were run to eliminate the signal of possible interfering compounds from those of target PAHs. Laboratory vials and materials were cleaned with neutral detergents, then pure water, then technical acetone to avoid cross contamination.

## 3. Results

We explained in the introduction how petroleum has been commonly used in pesticides, either as an ingredient in formulations, or as a pesticide in itself. This is an original review of the literature because it is generally not admitted that commercial pesticides, including even supposedly environmentally friendly ones, can contain petroleum products, and that this has been the case for centuries (Table 1). Some representative papers have been selected to demonstrate that this was well known and published as early as the 1780s and, in the 20th century, in each decade since 1950. In addition, we show that when petroleum products were evidenced in the early stages, petroleum or pesticides were also admitted to being carcinogenic or toxic (in bold in Table 1). For all these periods to date, in publications from researchers of the petroleum or agribusiness companies, petroleum has been and is recommended to be added to the formulations to improve the efficiency of the pesticides (Table 1).

In this work, we have investigated herbicides for gardeners or farmers during the latter decades of the 20th century up to now (Table 2). Since PAHs were discovered in all available recent common garden herbicides [23] we wondered if these could be detected in older ones, even if their presence was not indicated and they were not tested for long-term toxicity in mammals. For full knowledge, Table 2 recalls all the marketing specifications or labels of the pesticides that have been studied over time, whatever the declared supposedly active ingredient or the company (Holder). It is noticeable that the percentage of the chemical compound taken as the pesticide varies from only 0.92% (Roundup 6H) to a maximum of 71.7% (Target) of the total composition, as written on the label of the container. The totality of the ingredients is being ultimately sprayed in the environment. This is in part the object of this study, in particular concerning PAHs, which are never declared as chemical components, though they are not biologically inert. To understand the variability of the composition of PAHs in pesticides, different lots were measured for the same product, which serve also as internal controls for the different methods and analyses (E1, E2, E3). All the analysed herbicides are referenced in Table 4, with their code names, from A to W.

**Figure 1:**
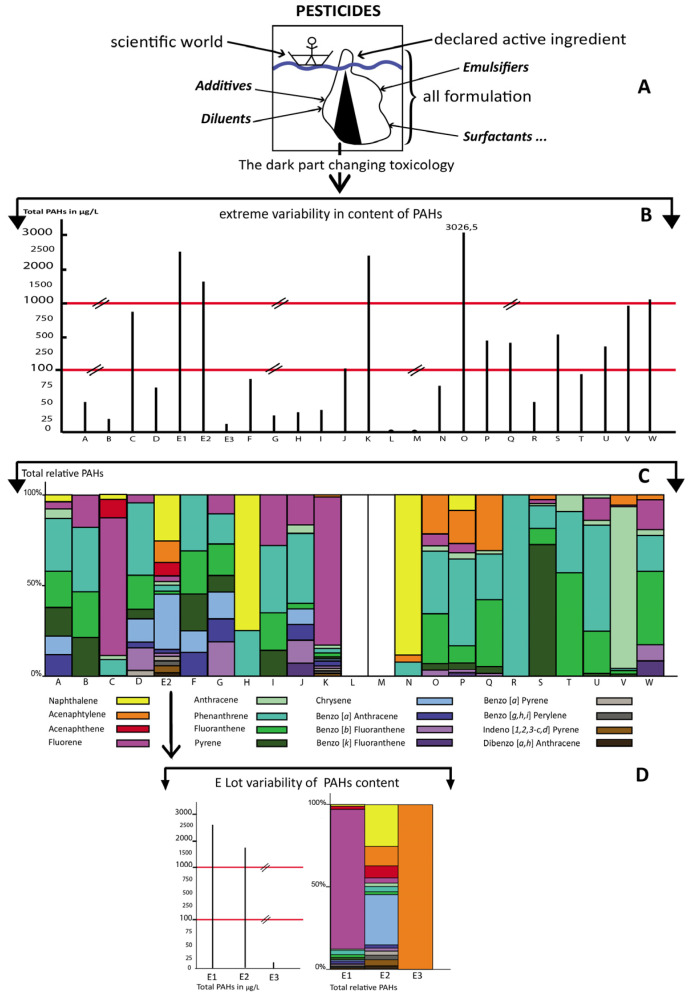
The hidden part of pesticide toxicology altered by formulants and contaminants. At the top (**A**), a symbolic iceberg shows the major role of formulants (additives, diluents, emulsifiers, surfactants, etc.) in pesticide toxicology. The extreme variability in content of PAHs (crude levels) is not taken into account, including percentage of the total formulation (**B**), since only the declared active ingredient is considered. The levels of PAHs in different lots of the same product appear variable and are not declared but studied in that work (**C**), including the variability for the same pesticide but different lots (**D**). L and M appear without any of the 16-priority PAHs measured; however, lighter monoaromatic hydrocarbons could be present (**C**).

**Figure 2:**
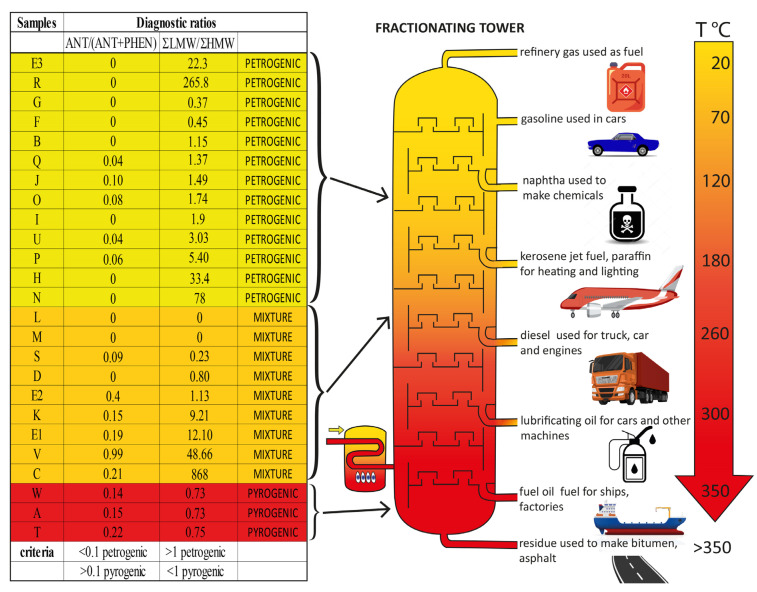
Fractionating tower of different petroleum products. On the left, a table shows, according to the samples, the petrogenic or pyrogenic ratio of the mixture found in the petroleum residues in formulants of pesticides in this study. The origin in the fractionating tower is indicated on the right, highlighting that the ratio can give different origins of PAHs.

In Table 4, E2 (Clairland, Table 2) appears, for instance, as one of the pesticides containing the highest concentrations of PAHs. The variability per lot (E2) is shown as an example in Figure 1 (bottom, D). All the 16 main PAHs commonly measured by regulatory environmental agencies, for air, water, soil or food pollution, are described by product in terms of quality, quantity (in µg/L), and standards or thresholds of toxicity, according to at least one of the national or international agencies involved in controls and regulations (Table 3). The structures, number of aromatic cycles, and molecular masses allow some classification of the PAHs. The other physicochemical characteristics, such as boiling points, vapour pressures, lipophilicity or hydrophilicity (Log Kow) for each PAH, as well as the aqueous solubility at 25 °C, indicate the properties that will be helpful to understand what kind of PAHs are present in pesticides (Table 3), and which part of refined petroleum they may represent, in particular in the petroleum distillation column (Figure 2). Numerous samples reach such high levels of PAHs that their ratio to the threshold of toxicity or carcinogenicity (recognized as a standard by international agencies in water) was extremely important, from 2.16 to 8288 times (last column, Table 3). Eleven PAHs out of the 16 were more than 50 times over the standards, and among these, 3 exceeded the standards by over 1000 times (Table 3).

The pesticides from A to W (Table 4), are detailed for the quantity of each measured PAH in µg/L. The first result evidences the great variability of the PAH concentrations in the different samples: from <2 µg/L (in D for example) to 2200 µg/L (for FLUO in E1). Maxima of the total of the 16 PAHs per pesticide (<10 µg/L for L or M to 3026.5 µg/L for O, Table 4) do not appear to be of the same order of magnitude. It even changes for each PAH in a given pesticide, and even for different lots of a same pesticide, from E1 to E3 for instance. This fact can be only partially explained by changes in the thresholds of detectability depending on the analytical method used, or by changes on the detergents found in each pesticide, which could possibly impact the detectability of PAHs. Benzalkonium chloride, for instance, is declared as an ingredient in herbicide N; it is a potent quaternary ammonium cationic detergent, very aggressive biologically. Detergents have a more or less intense foaming effect, as a function of their nature. Foam formation may possibly interfere with PAHs analyses and lead to possible losses during the analytical process. However, surrogate PAH standards were added during the analytical process for a majority of pesticides and did not show significant PAH losses (<9.6%, on average), whatever the nature and importance of detergent foaming. Moreover, if significant discrepancies could appear between lots (E1, E2 and E3), replicates were done on a majority of the analysed formulations (E3 and O to W) which did not show deviations between analyses of a same formulation.

The levels and distribution of PAHs per pesticide were noticeably also different. For instance, PHEN is found at 10, 71.1 or 216.7 µg/L in B, S and W formulations, respectively, even though they are from the same holding company (Table 3). By contrast, the distribution and levels of PAHs are not very different in the formulations E1 and K, which are from the same holding company but from different countries (France and Poland). Moreover, if most of the formulations contain PAHs mixtures, some of them contain only one PAH: R only contained PHEN and E3 only contained ACY (the threshold is different according to the method of measurement, but “<” indicates no detectable PAH). All these results show no homogeneity for these compounds in pesticide formulations. Petroleum residues appear to be more of a waste petroleum product than a real defined intentional ingredient.

In Figure 1, the total of the 16 PAHs are indicated in crude amounts from A to W, and they represent the hidden part of the iceberg of a pesticide formulation, where the declared active ingredient is the minor part. However, the declared active component is often wrongly taken as the full pesticide for toxicity calculations, such as the acceptable daily intake (ADI) or the toxic equivalent factor (TEF). The extreme variability of PAHs is represented in quantity. The PAHs are brought to 100% per pesticide to represent qualitatively the variability of the compounds, which is highlighted for lot E (E1, E2, E3). The LMW PAHs (2–3 cycles) are more represented in pesticides than HMW PAHs (4–6 cycles) which underlines that the petroleum residues appear to come mainly from crude unburned material. L and M contain no PAH; but it is theoretically possible that lighter monoaromatic hydrocarbons such as benzene, toluene or xylenes could be present, or other PAHs outside the 16 measured.

In Figure 2, it is obvious that low-ring PAHs are predominant in the analysed pesticide formulations. With the exception of formulations R and E3, where only one PAH could be quantified, a mixture of PAHs was present in all the other formulations. The majority of HMW PAHs were not detected in those mixtures, from B[*b*]FLT to B[*ghi*]PER. Only A, D, E1, E2, K, O, P, Q and W formulations contained low amounts of the 5-6-ring PAHs, such as B[*b*]FLT, B[*k*]FLT or B[*a*]PYR.

It must be emphasised that pesticide formulations were mainly aqueous. The solubility of HMW PAHs is extremely low in aqueous media, and only LMW PAHs are able to solubilize into water at low concentrations (Table 3). However, the studied pesticide formulations formed stable emulsions. In fact, PAHs are lipophilic, as can be shown by their high values of octanol/water partition coefficient (K_ow_) (Table 3). But emulsions, whether stabilized or not by additives such as so-called surfactants, for instance, benzalkonium chloride or polyoxyethylene amines POEA [31], consist of dispersions of lipophilic phases into water, or inversely, and allow insoluble compounds to solubilize into aqueous formulations. This is why some heavier PAHs, such as B[*b*]FLT or B[*a*]PYR, which are almost insoluble in water, could be found in some pesticide formulations.

Finally, it must be emphasised that NAPH, a lower-ring PAH, was rarely found in the studied formulations (E2, N and P for instance). But as indicated in Table 3, it has a markedly higher vapour pressure than the other PAHs, which makes it particularly volatile.

Concerning the origins of PAHs in different matrices, some authors chose to use a maximum of 2 diagnostic ratios to identify potential sources, in order to avoid contradictory interpretations [32]. Here, as HMW PAHs such as CHRYS, B[*a*]PYR, InPYR or B[*ghi*]PER were generally not present in the pesticide formulations, ΣLMW/ΣHMW and ANT/(ANT+PHEN) ratios were chosen to discriminate the pyrolytic or petrogenic sources of PAHs (Figure 2). As ΣLMW/ΣHMW ratio > 1 indicates a major petrogenic source, it appears that a majority of pesticides contain PAHs from unburned petroleum oil. However, F, G and S formulations were marked by ratios < 0.5, a strong pyrogenic PAH signature. 

The isomeric ratio ANT/(ANT+PHE) was < 0.1 for old pesticides (Table 3) such as O, P, Q, S and U formulations, which are characteristic of PAHs derived from petroleum that did not undergo high thermal processes. However, old T and W formulations had a ratio > 0.1, indicating that a part of the PAH mixture present in those formulations could come from heavier petroleum oils or potentially from burned petroleum, as mentioned previously. In fact, when this ratio is > 0.1, it means that PHEN is at least nine times more concentrated than ANT, as in petroleum fractions that have been subjected to high temperature or combustion treatments, where the less stable ANT has been transformed and disappeared. Thus, an increase in ANT over PHEN seems to show that PAHs could come from crude unburned petroleum, petroleum fractions that were not subjected to high temperature or combustion treatments. It is particularly the case for the V pesticide formulation, where ANT concentration was particularly high.

Figure 2 shows that the sources of PAHs in pesticide formulations as diagnostic ratios are sometimes contradictory. It is clear that crude petroleum oils have been introduced into a majority of pesticide formulations, but sometimes with a significant contribution of heavier petroleum oils (such as shale oil, for example) or burned oils.

## 4. Discussion

The pesticides in this study have not been assessed for their long-term toxicity as complete formulations, and they are not safe for two reasons. First, the scientific community does not know that petroleum products are generally used in pesticides, because they are not declared. Yet petroleum is a model substance used in the study of carcinogenicity and toxicity. Second, the declared active ingredients are tested purified and alone in the long-term toxicological evaluations submitted by industry to regulatory agencies, even though they are only minor parts of the constituents or at most half of the complete pesticide formulation. The ADI, for instance, should be divided by 1000 if it is considered that petroleum residues amplify the toxicity by 1000 [18,21,22,33,34].

If the presence of petroleum residues is intentional and neglected in the toxicity calculation of the commercial products, this could well conceal the real effects of the pesticides, in terms of the damaging impacts on the environment and health. Moreover, declaring the petroleum residues [24] could change all the regulatory toxicology results. In other words, the mixture might be highly carcinogenic or toxic in the long term, even more than the molecule of the declared active ingredient itself, with possible epigenetic consequences, thus affecting several generations at the human and all biosphere levels.

This has been possible only because the scientific world did not have any access to the complete formulations treated as industrial secrets. In a previous study, we found petroleum products in pesticides in newly commercialized herbicides without glyphosate [23]; thus, we investigated in this study the presence of PAHs in older herbicides, as well as their pyrogenic or petrogenic origin. It appeared that PAH mixtures present in the final pesticide formulations were intentional and that the petroleum moiety was not coming from accidental contaminations. But in fact, petroleum appears to have been used since the beginning of the history of pesticides [1], although it has been known to be toxic and carcinogenic for a long time [9,11]. This would correspond grossly to Benchmark Dose Lower bound (BMDL, EFSA) for B[a]PYR.

To know if, in a mixture of pesticides, the dominant composition comes from hydrocarbons having burned, it is indicative to calculate the ΣLMW/ΣHMW PAHs ratio and the ANT/ANT+PHEN ratio, which are complementary [32]. As ANT degrades more easily than PHEN at high temperatures, it tends to disappear if the mixture comes from burned petroleum. The examination of the ratio ΣLMW/ΣHMW shows that the PAHs found in a majority of samples are rather representative of crude oils and fluids or lighter refined petroleum cuts, such as kerosene, diesel or fuel oils [4]. So, it seems probable that PAHs present in the pesticide formulations originate more from the distillates obtained between 180 and 350 °C (Figure 2). Indeed, our results show that PAHs with lower boiling points could be present in distillates that generate paraffins (180–220 °C), diesel (200–260 °C), fuel oil (260–350 °C) or lubricating oil (300–350 °C). HMW PAHs, from FLT to DB[*a*]ANT, are generally present only in heavier distillates that generate bitumen (>350 °C). But it appears from this study that recent pesticide formulations could also contain the heaviest distillates, which in turn contain the more toxic PAHs. NAPH has a markedly higher vapour pressure than the other PAHs and it is possible that it was lost by volatilisation, as the cans of pesticide formulations tested were old, with some being almost forty years old.

## 5. Conclusions

The sale of pesticide substances to people without serious control has undoubtedly generated since the second world war a large number of deaths for diseases related to endocrine and nervous disorders [35], malformations, and cancers. Among the diseases linked to pesticides in which petroleum is involved are in particular autism, psychiatric diseases, reproductive pathologies, and a decline in fertility [2,36,37].

Investigations of the externalities of petroleum chemistry for pesticides with regard to climate change and public health should also take these data into account when the presence of petroleum is demonstrated in a pesticide.

This calls for a complete revision of regulatory toxicology with at least a 1000-fold decrease in the ADI, which is today based only on the active ingredient, if the petroleum residues, not considered to date, amplify the toxicity of all pesticides by 1000.

A study of the health of the users of these products would be pertinent, as part of an environmental forensic investigation of this problem.

## Figures and Tables

**Table 1:** Use of petroleum oil either as a pesticide since 1787, or within pesticides in each decade of the latter half of the 20th century. In bold: first instances of knowledge of the carcinogenicity or toxicity of petroleum or pesticides.

Year	Group	Comment on Petroleum Oil Products:	References
1787	Colonial pesticides information, UK	Used as an insecticide as long ago as 1787	Goeze [7], cited in Colwill [5], 1957
1950	Shell Research, UK	Insecticidal from 0.1–3% at least	Eaton and Davis [8]
1953	National Cancer Institute USA	**Crude and processed oils possess carcinogenic properties**	Hueper [9]
1954	Esso Standard Oil USA	Excellent solvent for pesticide formulations	Nelson and Fiero [1]
1957	Colonial Pesticides Information Service UK	Frequently employed with insecticidal and fungicidal agents–herbicidal activity increases with the aromaticity of the oil–solvent carriers of pesticides	Colwill [10]
1965	**USA Public Health Service**	**Carcinogenic potential of pesticides**	Falk et al. [11]
1967	Shell Research Ltd. UK	Petroleum blended with pesticides	Eaton [12]
1971	University of Moscow, USSR	The successful use of pesticides depends to a large degree on the formulation, mainly with petroleum products	Melnikov [6]
1980	University of London, UK	The addition of oil gives greater efficiency of active ingredient	Wodagenesh [13]
1990	American Society for Testing and Materials, Philadelphia, USA	Petroleum solvents are used as inerts in pesticide formulations	Curcio [14]
1990	Ohio State University USA	Horticultural (petroleum) oils in combination with insecticides have been used for decades	Nielsen [15]
2002	Forest Research Australia	Enhancement of pesticide activity by oil adjuvants **Necessity of global evaluation**	Zabkiewicz [16]
2002	Agricultural Experiment Station, New York USA	Petroleum distilled oils used for pest control over a century	Agnello [17]
2005	University of Caen Normandy, France	Toxicity and endocrine disruption of glyphosate amplified at least 100 times by formulations	Richard et al. [18]
2006	Texas AgriLife Extension, USA	Oils used as pesticides for centuries, include distillation products from petroleum	Bogran et al. [19]
2011	Toxicology Argentina	Mode of action of petroleum oils as pesticides	Buteler and Stadler [20]
2013	University of Caen Normandy, France	Polyethoxylated-petroleum derived products toxic in pesticides	Mesnage et al. [21]
2016	University of Caen Normandy, France	Endocrine disruption and human cell toxicity by pesticide co-formulants	Defarge et al. [22]
2020	University of Caen Normandy, France	Oil residues in herbicides without glyphosate	Seralini and Jungers [23]
2022	European Union	Petroleum is a co-formulant of pesticides	EFSA [24]

**Table 2:** Samples of herbicides used to measure PAHs in this study and dates of production.

Sample	Herbicide Name	Made Date	Declared Ingredient	%	Authorization	Holder	Provider	Lot Number
A	Roundup Speed-Evergreen Monsanto	2018	Acetic acid	6	2130153	Monsanto technology LLC	Evergreen Garden Care France SAS (69)	C8N515
B	Fertiligene-Herbatak Contact Scotts	2018		6	2130153	SCOTTS France SAS	SCOTTS FRANCE SAS (69)	338 18 07:16L23
C	Biocontrole Jardin d’Eden-Starnet Jade	2018	Pelargonic acid	51.9	2170243 n°CAS 112-05-0	JADE	START (37) JE_DBIO250	V050CB
D	Fertiligene-Herbatak Express Scotts Jade	2018		51.9	2170243	JADE	SCOTTS France	353 18 08:14 L25
E1	Clairland-Herbistop Compo	2018		24.3	2140121	COMPO France SAS	COMPO France SAS	21/11/2018/A
E2		2019						04/04/19/A
E3		2019						17/9/2019/A
F	Clairland express-Herbistop spray	2019		3.1	2160115	COMPO France SAS	COMPO France SAS	190508
G	Solabiol-Beloukha Garden	2019		51.9	2170243	JADE	SBM Life Sciences SAS	19031
H	Neudorff-Finalsan	2018		18.8	2170355 CAS 112-05-0	W.Neudorff GmbH KG	Or Brun (85)	11806086
I	Roundup-Unkrautfrei Germany	2019		51.92	Nr 008529-62	Belchim Crop Protection NV	Evergreen Garden Care Deutschland	NR.008529-62 1088/3285-CLP 12892398 C9N907
J	Target-Poland	2017		71.7	MRiRW nrR-140/2017	Belchim Crop Protection	Target SA	MriRWnrR-140/2017 z
K	Compo-Poland	2016		24.26	MRiRW nrR-34/2016	COMPO GmbH	COMPO Polska	MRiRW nr R-34/2016 wu z
L	Solabiol-Herbiclean	2018	Caprylic and Capric acids	3	2140167	SBM Développement SAS	SBM Life Sciences SAS	LOT 147MC38
M	Solabiol-Herbiclean	2015		1.8 + 1.2	2140167	SBM Développement SAS	SBM Life Sciences SAS	LOT 43135
N	Bros-Poland	2019	Benzalkonium Chloride	1.25	1000/04	BROS Sp. Zo.o. sp.k. Polska	BROS Sp. Zo.o. sp.k. Polska	DW/EXP 03 2022 UFI: 2JFA-40VN-G008-8JWH
O	Domodev	<2008	Glyphosate	36	9900028	Domodev	Domodev	
P	Burren	1985		36		Barclay chemicals LTD Dublin	BHS	
Q	Roundup 6H	<2000	Glyphosate + Pelargonic acid	0.72 + 0.204	2120157	Monsanto	SCOTT France SAS	C4001 B1001
R	KB desherbant liquide	<2010	Glufosinate	6	8900339	Hoechst	Rhone poulenc	0 05 V 136
S	Cora desherbant gazon	<2000	Mecoprop.P+2.4MCPA+Dicamba	20 + 10 + 2.4	9000662	SCOTTS France SAS	CORA	
T	Round up express	<2010	Glyphosate	7.2	2010321	Monsanto agriculture France SAS	SCOTT France SAS	C3029 B071P
U	Burren	<2010		36	2000499	Barclay chemicals LTD Dublin	BHS	PG-BN.448843.SEG
V	STARANE 200	<2010	Fluoroxypyr	20	8400600	DOW agro sciences SAS	DOW Agro sciences	
W	Likid allees	<2015	Glyphosate+Diflufenicanil	25	9800107	SCOTTS France SAS	Fertiligene	

**Table 3:** Physico-chemical properties of PAHs, toxicity standards and ratios of the maximum PAHs found in the bibliography [23,30].

Compounds/	Chemical Structure	Number of Cycles	Molecular Mass	Boiling Point (°C)	Vapour Pressure	Log K_ow_	Aqueous Solubility (25 °C) (mg/ L)	MaxPAHs	Toxicity PAHs Standards *	Max in	Max/ Standard *
*Abbreviations*			(g/mol)		(Pa at 25 °C)			(µg/L)	(µg/kg)	Samples *	
Naphthalene	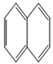	2	128.2	218	10.4	3.4	31.7	450	40, K	E2	11.25
*NAPH*											
Acenaphtylene	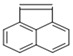	3	152.2	280	0.89	4.07	-	653.4	10	O	65.34
*ACY*											
Acenaphthene	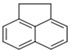	3	154.2	279	0.29	3.92	3.9	130	60	E2	2.16
*ACE*											
Fluorene	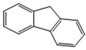	3	166.2	295	0.08	4.18	1.68	2200	40, K	E1	55
*FLUO*											
Anthracene	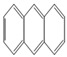	3	178.2	340	8.0 10^−4^	4.5	0.073	883.3	40	V	22.08
*ANT*											
Phenanthrene	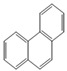	3	178.2	340	0.016	4.52	1.29	1046.7	20, K?	O	52.33
*PHEN*											
Fluoranthene	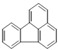	4	202.3	375	0.00123	5.20	0.26	828.8	0.1, K	O	8288
*FLT*											
Pyrene	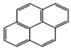	4	202.3	404	0.0006	5.18	0.135	411.5	30, K?	S	13.71
*PYR*											
Chrysene	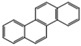	4	228.3	438	-	5.86	0.00179	540	50, K	E2	10.8
*CHRYS*											
Benz[*a*]	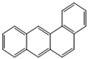	4	228.3	448	2.8 10^−5^	5.61	0.014	67	0.01, K	K	6700
Anthracene											
*B[a]ANT*											
Benzo[*b*]	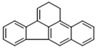	5	252.3	481	-	5.78	0.0015	97.9	0.1, K	O	979
Fluoranthene											
*B[b]FLT*											
Benzo[*k*]	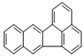	5	252.3	480	-	6.11	0.0008	91.4	1, K	W	91.4
Fluoranthene											
*B(k)FLT*											
Benzo[*a*]pyrene	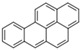	5	252.3	495	7.3 10^−7^	6.50	0.004	45	0.01, K	E2	4500
*B[a]PYR*											
Benzo[*g,h,i*]	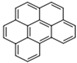	6	276.3	536	1.4 10^−8^	7.10	0.00026	48	0.1, K	E2	480
Perylene											
*B(ghi)PER*											
Indeno[1,2,3-*cd*]	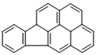	6	276.3	524	-	-	0.00019	68	0.2, K	E2	340
Pyrene											
*InPYR*											
Dibenz[*a,h*]	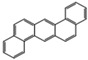	5	278.4	550	-	6.75	0.00050	33	0.5, K	E2	66
Anthracene											
*DB[ah]ANT*											

*: Max maximum level observed in this study in the samples indicated; Toxicity: toxicity standard set by the product determined by at least one national or international agency, including AFSSA, ANSES, EPA, INERIS, NIH, WHO. K: carcinogen, K?: probable or possible carcinogen; max/standard ratio of the maximal level to the toxicity standards.

**Table 4:** PAHs amounts for each pesticide A-W in µg/L. E1 to E3 were the same pesticide, but from different lots; they were used as internal controls in our blinded measurements. All values < mean < LOQ.

Compounds/	A	B	C	D	E1	E2	E3	F	G	H	I	J	K	L	M	N	O	P	Q	R	S	T	U	V	W
*Abbreviations*																									
Naphthalene	2.1	<5	24.0	<2	11.0	450.0	<22	<10	<2	25.0	<2	<2	<10	<10	<10	69.0	<22	42.3	<4.4	<22	<2.2	<2.2	<2.2	<2.2	<22
*NAPH*																									
Acenaphtylene	<5	<5	<15	<2	<10	210.0	22.3	<10	<2	<5	<2	<2	10.0	<10	<10	<3	653.4	88.8	131.1	<36.5	15.3	<3.7	<3.7	54.9	30.8
*ACY*																									
Acenaphthene	<5	<5	88.0	<2	29.0	130.0	<3.1	<10	<2	<5	<2	<2	<2	<10	<10	3.0	<31	<6.2	<6.2	<31	<3.1	<3.1	7.5	<3.1	<31
*ACE*																									
Fluorene	2.2	5.0	660.0	3.3	2200.0	54.0	<3.4	<10	3.3	<5	12.0	21.0	2100.0	<10	<10	<3	197	25.2	<6.8	<34	11.7	<3.4	50.6	2.2	179.4
*FLUO*																									
Anthracene	2.9	<5	20.0	<2	16.0	37.0	<35.5	<10	<2	<5	<2	6.0	11.0	<10	<10	<3	91.1	16.5	4.9	<35.5	7.2	8.8	10.5	883.3	34.2
*ANT*																									
Phenanthrene	16.0	10.0	76.0	30.0	68.0	56.0	<27.5	26.0	5.3	8.4	16.0	49.0	71.0	<10	<10	6.0	1046.7	238.9	109.6	265.8	71.1	31.9	240	8.5	216.7
*PHEN*																									
Fluoranthene	11.0	7.1	<15	14.0	46.0	30.0	<23.5	20.0	5.5	<5	8.9	3.8	55.0	<10	<10	<3	828.8	46.1	157	<23.5	50.7	53.9	95.6	18.9	442.4
*FLT*																									
Pyrene	8.7	6.0	<15	4.0	33.0	<15	<34	17.0	2.9	<5	6.1	<2	26.0	<10	<10	<3	111.6	18.1	17.8	<34	411.5	<3.4	6.2	0.6	<34
*PYR*																									
Chrysene	5.6	<5	<15	9.6	19.0	540.0	<81	10.0	4.7	<5	<2	11.0	28.0	<10	<10	<3	<81	<16.2	<16.2	<81	<8.1	<8.1	<8.1	<8.1	<81
*CHRYS*																									
Benz[*a*]	<5	<5	<15	2.5	39.0	35.0	<88	11.0	4.00	<5	<2	11.0	67.0	<10	<10	<3	<88	<17.6	<17.6	<88	<8.8	<8.8	<8.8	<8.8	<88
Anthracene																									
*B[a]ANT*																									
Benzo[*b*]	<5	<5	<15	9.3	17.0	32.0	<133	<10	6.00	<5	<2	16.0	22.0	<10	<10	<3	97.9	5.5	4.3	<133	<13.3	<13.3	<13.3	<13.3	97.6
Fluoranthene																									
*B(b)FLT*																									
Benzo[*k*]	<5	<5	<15	<2	<10	<15	<154	<10	<2	<5	<2	9.1	<10	<10	<10	<3	<1540	6.6	<30.8	<154	<15.4	<15.4	<15.4	<15.4	91.4
Fluoranthene																									
*B(k)FLT*																									
Benzo[*a*]pyrene	6.4	<5	<15	2.3	16.0	45.0	<409	<10	<2	<5	<2	<2	17.0	<10	<10	<3	<409	<81.8	<81.8	<409	<40.9	<40.9	<40.9	<40.9	<409
*B(a)PYR*																									
Benzo[*g,h,i*]	<5	<5	<15	<2	11.0	48.0	<1500	<10	<2	<5	<2	<2	12.0	<10	<10	<3	<1500	<300	<3 00	<1500	<150	<150	<150	<150	<1500
Perylene																									
*B(ghi)PER*																									
Indeno[1,2,3-*cd*]	<5	<5	<15	<2	11.0	68.0	<1800	<10	<2	<5	<2	<2	11.0	<10	<10	<3	<1800	<360	<360	<1800	<1800	<180	<180	<180	<1800
Pyrene																									
*InPYR*																									
Dibenz[*a,h*]	<5	<5	<15	<2	<10	33.0	<1800	<10	<2	<5	<2	<2	<10	<10	<10	<3	<1800	<360	<360	<1800	<180	<180	<180	<180	<1800
Anthracene																									
*DB[ah]ANT*																									
Total PAHs	54.9	28.1	868.0	75.0	2516.0	1768.0	22.3	84.0	31.7	33.4	43.0	126.9	2430.0	<10	<10	78.0	3026.5	488.0	424.7	265.8	567.5	94.6	410.4	968.4	1092.5

## Data Availability

Not applicable.

## Abbreviations

ACE: Acenaphthene; ACY, Acenaphthylene; ADI, acceptable daily intake; ANT, Anthracene; B[*a*]ANT, Benz[*a*]Anthracene; B[*b*]FLT, Benzo[*b*]Fluoranthene; B[*k*]FLT, Benzo[*k*]Fluoranthene; B[*a*]PYR, Benzo[*a*]Pyrene; B[*ghi*]PER, Benzo[*g,h,i*]Perylene; CHRYS, Chrysene; DB[*a,h*]ANT, Dibenz[*ah*]Anthracene; FLUO, Fluorene; FLT, Fluoranthene; HC hydrocarbon; HMW, High molecular weight; IS, Internal standards; InPYR, Indeno[*1,2,3,c,d*]Pyrene; LLE, Liquid-liquid extraction; LMW, low molecular weight; MS, Mass spectrometry; NAPH, Naphthalene; PAH, polycyclic aromatic hydrocarbon; PHEN, Phenanthrene; PTFE, Polytetrafluoroethylene; PYR, Pyrene; SS, Surrogate standards; TEF, toxic equivalent factor; US-EPA, United State Environmental Protection Agency.
